# Canadian Highly Sensitized Patient Program Report: A 1000 Kidney Transplants Story

**DOI:** 10.1177/20543581241306811

**Published:** 2024-12-24

**Authors:** M. Khaled Shamseddin, Steven Paraskevas, Rahul Mainra, Kyle Maru, Bailey Piggott, Darlene Jagusic, Kathy Yetzer, Lakshman Gunaratnam, Christine Ribic, Joseph Kim, Sunita Singh, Stephanie Hoar, G. V. Ramesh Prasad, Melanie Masse, Isabelle Houde, Myriam Khalili, Kenneth West, Rob Liwski, Sean Martin, Nessa Gogan, Martin Karpinski, Mauricio Monroy-Cuadros, Sita Gourishankar, Olwyn Johnston, James Lan, Christopher Nguen, John Gill, Michel Pâquet

**Affiliations:** 1 Division of Nephrology, Department of Medicine, Queen’s University, ON, Canada; 2 Department of Surgery, McGill University, Montreal, QC, Canada; 3 Division of Nephrology, Department of Medicine, University of Saskatchewan, Saskatoon, Canada; 4 Organ and Tissue Donation and Transplantation, Canadian Blood Services, Ottawa, ON, Canada; 5 Division of Nephrology, Department of Medicine, Western University, London, ON, Canada; 6 Division of Nephrology, Department of Medicine, McMaster University, Hamilton, ON, Canada; 7 Division of Nephrology, Department of Medicine, University Health Network, University of Toronto, ON, Canada; 8 Division of Nephrology, Department of Medicine, University of Ottawa, ON, Canada; 9 Division of Nephrology, Department of Medicine, St. Michael’s Hospital, University of Toronto, ON, Canada; 10 Division of Nephrology, Department of Medicine, University of Sherbrooke, QC, Canada; 11 Division of Nephrology, Department of Medicine, Laval University, Quebec City, QC, Canada; 12 Division of Nephrology, Department of Medicine, Montreal University, QC, Canada; 13 Division of Nephrology, Department of Medicine, Dalhousie University, Halifax, NS, Canada; 14 Department of Pathology and Laboratory Medicine, Dalhousie University, Halifax, NS, Canada; 15 Division of Nephrology, Department of Medicine, Memorial University, St. John’s, NL, Canada; 16 Saint John Regional Hospital, Horizon Health Network, NB, Canada; 17 Section of Nephrology, Department of Medicine, University of Manitoba, Winnipeg, Canada; 18 Division of Transplant Surgery, Department of Surgery, University of Calgary, AB, Canada; 19 Division of Nephrology and Transplant Immunology, Department of Medicine, University of Alberta, Edmonton, Canada; 20 Division of Nephrology, Department of Medicine, The University of British Columbia, Vancouver, Canada; 21 Department of Urological Sciences, The University of British Columbia, Vancouver, Canada; 22 Division of Nephrology, Department of Medicine, Centre Hospitalier de l‘Université de Montréal, QC, Canada

**Keywords:** highly, sensitized, patients, kidney, transplant

## Abstract

**Purpose:**

Highly sensitized patients (HSPs) with kidney failure have limited access to kidney transplantation and poorer post-transplant outcomes. Prioritizing HSPs in kidney allocation systems and expanding the pool of deceased donors available to them has helped to reduce their wait times for transplant and enhanced post-transplant outcomes. The Canadian HSP Program was established by Canadian Blood Services in collaboration with provincial organ donation and transplantation programs throughout the country to increase transplant opportunities for transplant candidates needing very specific matches from deceased kidney donors. Highly sensitized patients in the Canadian Program are defined by a calculated panel-reactive antibody (cPRA) ≥95%. In this report, we describe the evolution and trajectory of the Canadian HSP Program and evaluate the national impact on the first 1000 kidney transplant cases.

**Source of Information:**

To allocate deceased donor kidney organs nationally to HSPs and report on the Canadian HSP Program’s performance, Canadian Blood Services developed a national database registry known as the Canadian Transplant Registry (CTR) and an online reporting tool known as the Canadian HSP Program Data Dashboard.

**Methods:**

The CTR, which collects HSPs’ data for the purpose of matching potential donors to HSPs and as part of required national quality, safety, and efficiency performance measurements, was retrospectively reviewed. Due to the nature of using deidentified aggregate registry data, a patient consent form was not required. A Research Ethical Board (REB) application was also waived.

**Key Findings:**

In this article, we describe the historical development, initial deployment, and evolution of the Canadian HSP Program with a primary aim to increase the rate of deceased donor kidney transplantation. A secondary aim was to evaluate the national impact of the Canadian HSP Program on the first 1000 kidney transplant cases. Transplant candidates who have participated in the Canadian HSP Program and recipients who received transplants were predominantly females (average age 50 years, female 62%) with blood group O (47% of candidates, 42% of transplants). Seventy percent of all active transplant candidates enrolled in the HSP Program were in the hardest to match group (cPRA ≥99%), and only 22% of the transplant candidates with cPRA of 100% have received a transplant to date through the Program. The average times from first participation in the Canadian HSP Program to transplantation for cPRA ≥99% transplant recipients were significantly longer than for cPRA 95% to 98% recipients averaging 22 months versus 6 months, respectively. By the end of June 2024, the Canadian HSP Program had facilitated 1000 transplants, 613 of which were from interprovincial matches. The average (SD) cold ischemic time (CIT) was 14.5 (5.9) hours, with interprovincial transplants exhibiting significantly longer CITs compared with intraprovincial transplants, averaging an additional 4.7 hours.

**Limitations:**

Our study limitations include first that it is a retrospective registry data analysis with no available short- and long-term clinical outcomes data at this point (patient and graft survival). Second, given the nature of registry data, not all relevant data may have been captured and reporting may not be complete for all patients.

**Implications:**

Examination of CTR registry data showed the Canadian HSP Program had a meaningful impact in enabling 1000 HSPs to access transplantation opportunities that may otherwise be unavailable to them.

## Abrégé

**Contexte:**

Les patients hyperimmunisés (PHI) atteints d’insuffisance rénale ont un accès limité à la transplantation et présentent de moins bons résultats après la greffe. La priorisation des PHI dans les systèmes d’allocation de reins et l’élargissement du bassin de donneurs décédés à leur disposition ont permis de réduire le temps d’attente de ces patients pour la transplantation et d’améliorer leurs résultats post-greffe. Le Programme canadien des PHI (PPHI) a été développé par la Société canadienne du sang en collaboration avec les programmes provinciaux de don et de transplantation d’organes de partout au pays afin d’accroître les possibilités de greffe pour les candidats qui ont besoin d’une compatibilité très spécifique avec des donneurs de rein décédés. Les PHI du Programme canadien sont définis comme des patients dont le PRAc (anticorps réactifs contre les échantillons du panel calculé) est d’au moins 95 %. Dans ce rapport, nous décrivons l’évolution et la trajectoire du PPHI et nous évaluons l’impact national des 1 000 premiers cas de transplantation rénale.

**Sources de l’information:**

Afin d’attribuer les reins de donneurs décédés aux PHI à l’échelle nationale et de rendre compte du rendement du PPHI, la Société canadienne du sang a créé un registre de base de données national, le Registre canadien de transplantation (RCT), et un outil de déclaration en ligne, le Tableau de bord des données du PPHI.

**Méthodologie:**

Le RCT, qui recueille les données des PHI dans le but de les jumeler à des donneurs potentiels, a fait l’objet d’un examen rétrospectif dans le cadre des mesures nationales requises en matière de qualité, de sécurité et d’efficacité. En raison de l’utilisation d’un ensemble de données de registre dépersonnalisées, un formulaire de consentement du patient n’était pas requis. Il n’a pas non plus été nécessaire de demander l’avis du comité d’éthique de la recherche (CER).

**Principaux résultats:**

Dans cet article, nous décrivons le développement, le déploiement initial et l’évolution du PPHI dans l’objectif premier d’augmenter le taux de transplantation rénale avec donneurs décédés. Un objectif secondaire était d’évaluer l’impact du PPHI à l’échelle nationale sur les 1 000 premières transplantations rénales. Les candidats à la transplantation qui ont participé au PPHI et qui ont reçu une greffe étaient majoritairement des femmes (62 %; âge moyen: 50 ans) de groupe sanguin O (47 % des candidats; 42 % des greffés). De tous les candidats à la transplantation actifs qui étaient inscrits au PPHI, 70 % faisaient partie du groupe le plus difficile à jumeler (PRAc ≥ 99%) et seulement 22 % des candidats à la transplantation avec un PRAc de 100 % ont reçu une greffe à ce jour dans le cadre du Programme. Pour les receveurs avec un PRAc ≥99 %, les délais moyens entre la première participation au programme et la transplantation étaient significativement plus longs que pour les receveurs avec un PRAc de 95 à 98 % (moyenne de 22 mois c. 6 mois). À la fin de juin 2024, le PPHI avait facilité 1 000 transplantations, dont 613 jumelages interprovinciaux. Le temps moyen (écart-type) d’ischémie froide était de 14,5 (5,9) heures, les greffes interprovinciales présentant des périodes d’ischémie froide significativement plus longues que les greffes intraprovinciales, soit de 4,7 heures plus longues en moyenne.

**Limites:**

Notre étude est limitée par le fait qu’il s’agit d’une analyse rétrospective des données de registres sans données disponibles sur les résultats cliniques à court et à long terme à ce stade (survie du patient et du greffon). Aussi, compte tenu de la nature des données de registre, il est possible que toutes les données pertinentes n’aient pas été saisies et que les rapports ne soient pas complets pour tous les patients.

**Conclusion:**

L’examen des données du RCT a montré que le Programme canadien des PHI avait eu un impact significatif en permettant à un millier de patients hyperimmunisés d’accéder à des possibilités de transplantation qui pourraient autrement leur être indisponibles.

**Connaissances à ce jour:**

Les patients hyperimmunisés (PHI) atteints d’insuffisance rénale ont un accès limité à la transplantation rénale et présentent de moins bons résultats post-transplantation. Améliorer la transplantation rénale pour les PHI est un défi mondial. Partout dans le monde, le fait d’accorder une priorité aux PHI dans les systèmes d’allocation des reins a permis d’augmenter les possibilités de transplantation, de réduire les temps d’attente et d’améliorer les résultats post-transplantation.

**Ce que ce document de recherche ajoute:**

Le Programme canadien des PHI (PPHI), développé par la Société canadienne du sang en collaboration avec les programmes provinciaux de don et de transplantation d’organes, a facilité avec succès les greffes de rein pour les PHI canadiens qui ont besoin d’une compatibilité très spécifique avec des donneurs de rein décédés. Ces patients n’auraient peut-être jamais reçu une greffe de rein par l’entremise de listes d’attente provinciales plus petites. Le partage du bassin de donneurs à l’échelle nationale par l’entremise du PPHI a entraîné une augmentation du nombre de greffes rénales chez les receveurs qui sont les plus difficiles à jumeler.

## Introduction

Sensitization to non-self human leukocyte antigen (HLA), which occurs primarily through previous blood transfusions, pregnancies, and/or prior transplants, limits patients’ access to kidney transplantation.^1-3^ High levels of donor-specific HLA antibodies (DSAs) present at transplantation are associated with a high risk of hyperacute rejection and worse graft outcomes.^4,5^

In the mid-1960s, complement-dependent cytotoxicity (CDC) crossmatch was the gold standard measure of HLA Abs, detecting only complement-activating DSAs.^6^ Flow cytometric crossmatch (FCXM) was developed later in the early 1980s^7,8^ to additionally detect non-complement fixing DSAs associated with graft rejection and loss.^9^ In the early 2000s, more sensitive solid-phase assays^10,11^ using single antigen beads (SAB) were developed to detect and identify specific HLA Abs in potential recipients.^12,13^ The degree of HLA Ab binding to a specific bead is expressed as mean fluorescence intensity (MFI), which identifies by virtual cross match (VXM) sensitized patients^14^ with potentially unacceptable HLA mismatches that should be avoided when selecting a donor.^15^ The ability of DSAs identified by SAB to bind donor cells ex vivo in FCXM (DSA+/FCXM+) at the time of transplantation is a strong predictor of subsequent antibody mediated rejection (ABMR) and graft loss, occurring in 50% and 30% of recipients, respectively, within 5 years post-transplant.^6,12,16,17^

Highly sensitized patients (HSPs) have been defined by a panel-reactive antibody (PRA) value of 70% to 100% depending on the organ donation organization (ODO).^6,13,18^ Achieving successful kidney transplant for HSPs is a global challenge. Highly sensitized patients on the kidney waitlist have the least chance to receive a crossmatch-negative donor offer, and once they have a transplant, have lower patient and graft survival than non-HSPs.^18^ Thus, kidney transplant candidates with a PRA >80% received additional allocation points in the United Network for Organ Sharing (UNOS) allocation system to compensate for their biological disadvantage for finding a match.^19^

Later in 2009, calculated PRA (cPRA) computed from HLA antigen frequencies among approximately 12 000 kidney donors in the United States between 2003 and 2005 was used to award sensitization points and better donor kidney allocation to HSPs.^19^ With 90% concordance with a PRA ≥80% and a total calculation of both class I and II HLA specificities, cPRA replaced peak and current PRA providing a more accurate estimate of sensitization.^19^ Depending on markedly reduced transplantation rates compared with less sensitized patients, patients with a cPRA ≥95% were considered HSPs, while those with a cPRA ≥99% were identified as very HSPs.^20^

## Priority Points in Regular Allocation

In December 2014, the new Kidney Allocation System (KAS) resulted in increased transplant rates for sensitized patients in the United States by using a sliding scale point system that will incrementally award bonus points on an exponential scale to patients with a cPRA >20%.^21^ Patients with a cPRA of 99% receive further priority by regional allocation while those with a cPRA of 100% benefit from national allocation.^21-23^ Consequently, the transplant rate has increased for HSPs from 2.4% to 13.4% in the first year of implementation,^24^ while the median waiting time decreased from >19 to 3.2 years.^25^ Meanwhile, shipping organs over longer distances increased cold ischemic times (CITs). The proportion of shipped organs with CIT >24 hours has increased during the first year after KAS implementation from 21.4% to 29.2% and was associated with higher risk of delayed graft function (DGF),^24^ although 6-month graft survival was not affected. In addition, the rate of zero HLA (A, B, and DR) mismatches significantly declined in the new KAS, which will likely increase the risk of HLA immunization^24^ and potentially lead to poorer graft survival with no available long-term data.^24,25^

## The Acceptable Mismatch Programs

Regular deceased donor allocation is based on avoidance of unacceptable HLA mismatches (ie, HLA antigens for which a patient has DSA). Allocation can also be adjusted, for the increased waiting time of HSPs, by the development of an Acceptable Mismatch (AM) program allowing matching to donors with HLA antigens to which the patient has made no proven SAB-based HLA Abs.^26^ Acceptable HLA antigens are added to create a patient’s “extended HLA type” on which matching is performed.^26^

The Eurotransplant AM program has enabled successful transplantation with significantly decreased waiting times and excellent outcomes for HSPs with a cPRA >85% who had been on dialysis for at least 2 years.^27^ Although transplantation did not increase significantly for HSPs, especially those with cPRA >99% (25% of listed HSPs have <0.015% chance to match a donor), HSPs who received a transplant through the AM program had a significantly better 10-year graft survival compared with patients who received a transplant through regular allocation (72.8% vs 62.4%).^27^ Death-censored graft survival rate in HSPs was also similar to non-sensitized patients related to a lower risk of rejection.^28^

Simulations done on the EUROSTAM project, initiated in 2012, have shown that expanding the deceased donor pool by sharing donor kidneys between European countries and registries will increase compatible transplant offers by 27% among 724 HSPs with cPRA >95% who had been registered for at least 5 years.^29^

## Canadian HSP Program

Approximately 20% of patients on provincial waitlists^30^ are highly sensitized, defined by having a cPRA ≥95%. Historically, and prior to the Canadian HSP Program, Canadian HSPs receive <1% of available organs.^30^ With access to a limited number of donors in their home province, Canadian HSPs waited much longer for a kidney transplant and had a greater chance of becoming more ill or dying while on the waitlist.

By providing access to donors across the country, the Canadian HSP Program increases the chances of finding kidney transplants for these hard-to-match patients. The Canadian HSP Program was established by Canadian Blood Services in collaboration with provincial and territorial governments and organ donation and transplantation programs across the country to increase transplant opportunities for patients needing very specific matches from deceased kidney donors. Through the Canadian HSP Program, this group of patients now has access to a larger national donor pool, increasing the chance of finding a donor and receiving a compatible kidney transplant. The implementation of this national program began in October 2013 and by November 2014 all provinces and territories had joined the Canadian HSP Program.

Based on a goal set up by the working group to achieve 50 interprovincial transplants in the first year of the HSP program, the algorithm architect completed many simulations using different cPRA levels to be able to determine what cPRA would meet this number of transplants. Derived from simulations, it was determined that a cPRA of ≥95% would likely provide the number of transplants to meet the working group’s goal. During the first year of the Canadian HSP Program, the registry was able to complete 49 transplants.

Canadian Blood Services developed a national web-based computer program and database registry known as the Canadian Transplant Registry (CTR) which identifies kidney transplant opportunities across Canada for HSPs. Matching algorithm policies are used to identify and rank HSPs who are potential matches to an available donor kidney. In addition, the CTR generates value in terms of quality, safety, and efficiency through its capabilities to collect and analyze descriptive and clinical outcomes.

The HSP algorithm utilizes 3 categories in the matching process to generate a potential recipient for a deceased donor registered in the CTR. The categories are as follows: blood group compatibility, HLA compatibility, and recipient and transplant program-specific filters, including, but not limited to, donor’s age, HBV and HCV serologies, and death determination by circulatory criteria (DCC) status. In cases where more than one recipient matches to the registered donor, a ranking list is reviewed by the algorithm to create a ranked list of matching recipients for the organ offer. If the offer of the organ for the first-ranked recipient is declined, an offer can be made to the second-ranked recipient (Table S1).

## Interprovincial Organ Sharing

As part of its goal of making transplantation accessible to transplant candidates who are the hardest to match, the Canadian HSP Program enables transplant opportunities for these candidates from donors across Canada. The program defines an interprovincial transplant as one in which the donor’s organ ODO is in a different province from the province of the recipient’s provincial health card number, with the exception of the Atlantic provinces for which a transplant involving both a donor and a recipient from within that region are considered intraprovincial.

Prior to the Canadian HSP Program, kidneys from deceased donors stayed within the province of origin. Now, when there are 2 transplantable kidneys from a single deceased donor anywhere in Canada, one kidney is allocated through the CTR to the Canadian HSP Program for matching to potential recipients across the country, while the other kidney stays locally. Kidneys are shared between provinces using the CTR which tracks all HSP matches, offers, and transplants.

Since the implementation of the Canadian HSP Program, successful deceased donor kidney transplants have improved the lives of more than 1000 Canadians with kidney failure who would have faced the greatest difficulty in finding a compatible kidney transplant through their respective provincial transplant programs.

## Methodological Notes

The CTR is a Canadian patient registry, which collects data on HSPs as part of required national quality, safety, and efficiency performance measurements. This examination relays aggregate results that are consistent with program reporting requirements and are suitable for public consumption. The deidentified nature of the results reported here are within the scope of patient consents obtained in relation to participation in the Canadian HSP Program. A Research Ethical Board (REB) application was also waived. Details regarding the tests of statistical significance that were performed and support the claims made in this examination were carried out as part of reporting requirements for the Kidney Transplant Advisory Committee. Test results have been omitted here but may be made available on request.

Data as extracted from the CTR on July 17 and 18 of 2024 using standard program reporting methods and tools with results reflecting patient participation as of June 26, 2024. It should be noted that, although the accuracy of the data extraction process is regularly validated and steps are taken to address known errors in specific values, Canadian Blood Services does not independently perform systematic validations of data provided by partnering organizations (ODOs and/or transplant programs) in relation to patient information. However, as part of Canadian Blood Services regular communications with the stakeholders involved in the Canadian HSP Program, analytics are routinely performed. Where applicable, the results of these internal analytics have been included here when discussing key differences, specifically differences in time (cold ischemic time and transplant wait time) and the proportion of patients receiving transplants through the program.

Results by cPRA categorized by the most recent cPRA at Canadian HSP Program transplant (for transplant recipients) or the cPRA based on the most current serological testing available in CTR for candidates not receiving transplants. A total of 18 transplant candidates whose cPRA was revised to be below the eligible range for the program after their first participation have been excluded from these results. In total, 29 offers that were identified as being declined due to HLA issues were pending confirmation as of the creation of this report.

For the purposes of this analysis, the proportion of transplant candidates receiving transplants refers to the proportion of transplant candidates who participated in the Canadian HSP Program who received one or more transplants through the program. For patients receiving more than one transplant through the program (n = 10), time to second transplant is measured based on time from patient’s re-activation in the Canadian HSP program to the time of their second transplant.

When looking at differences in cold ischemic time and time on dialysis prior to transplant, the results for statistical significance were determined based on unpaired one-tailed *t*-tests with no control variables calculated through the statistical analysis utility in Microsoft Excel 2016. Based on the sampling frame of the first 1000 HSP transplants, time on dialysis was available for 970 cases and cold ischemic time was available for 651 records. In both cases, this statistical test presented ample power to identify significant differences and was robust enough to account for any skew/kurtosis issues. For the proportion of HSP program transplants going to recipients in the hardest-to-match category (cPRA 100%), the results were compared between time periods via a simple 2-by-2 crosstabulation using the online chi-square test calculator at www.quantpsy.org/chisq/chisq.htm. Expected frequencies were of sufficient magnitude that adjustments were not required.

These results are presented for reference and to contextualize the claims made in this work; further investigation of these topics that includes a more comprehensive analysis may be carried out as part of ongoing program reviews.

## Results and Key Findings

The average age of transplant candidates when they first participate in the Canadian HSP program is 50 years (Table 1), and the average age of transplant recipients is 51 years (Table 2). Sixty-two percent of the candidates and recipients are identified as female (Table 1 and 2). Forty-seven percent of all transplant candidates are blood group O (Table 1), and 42% of the transplants facilitated by the Canadian HSP Program have gone to blood group O recipients (Table 2).

**Table 1.** Age, Sex, and Blood Groups of Transplant Candidates Participating in the Canadian HSP Program.

cPRA	Total transplant candidates	Blood group: count (proportion within cPRA category)				Mean age (SD) at first participation	Proportion female
		A	AB	B	O		
95%	154	48 (31%)	5 (3%)	25 (16%)	76 (49%)	54 (13.9)	103 (67%)
96%	132	42 (32%)	9 (7%)	18 (14%)	63 (48%)	52 (14.5)	92 (70%)
97%	156	59 (38%)	4 (3%)	28 (18%)	65 (42%)	52 (13.9)	103 (66%)
98%	239	85 (36%)	6 (3%)	39 (16%)	109 (46%)	49 (14.0)	145 (61%)
99%	403	139 (34%)	13 (3%)	72 (18%)	179 (44%)	50 (13.5)	259 (64%)
100%	1224	380 (31%)	64 (5%)	178 (15%)	602 (49%)	49 (13.4)	720 (59%)
Total	2308	753 (33%)	101 (4%)	360 (16%)	1094 (47%)	50 (13.7)	1422 (62%)

**Table 2.** Age, Sex, and Blood Groups of Canadian HSP Program Transplant Recipients.

cPRA	Total transplant recipients	Blood group: count (proportion in cPRA category)				Mean age (SD) at transplant	Proportion female
		A	AB	B	O		
95%	111	36 (32%)	4 (4%)	18 (16%)	53 (48%)	55 (13.4)	75 (68%)
96%	107	37 (35%)	9 (8%)	15 (14%)	46 (43%)	53 (13.7)	72 (68%)
97%	111	42 (38%)	4 (4%)	22 (20%)	43 (39%)	52 (14.0)	78 (70%)
98%	149	62 (42%)	5 (3%)	25 (17%)	57 (38%)	51 (13.7)	87 (58%)
99%	249	94 (38%)	8 (3%)	44 (18%)	103 (41%)	51 (13.3)	158 (63%)
100%	273	93 (34%)	20 (7%)	46 (17%)	114 (42%)	49 (13.6)	152 (56%)
Total	1000	364 (36%)	50 (5%)	170 (17%)	416 (42%)	51 (13.6)	622 (62%)

**Figure 1.**
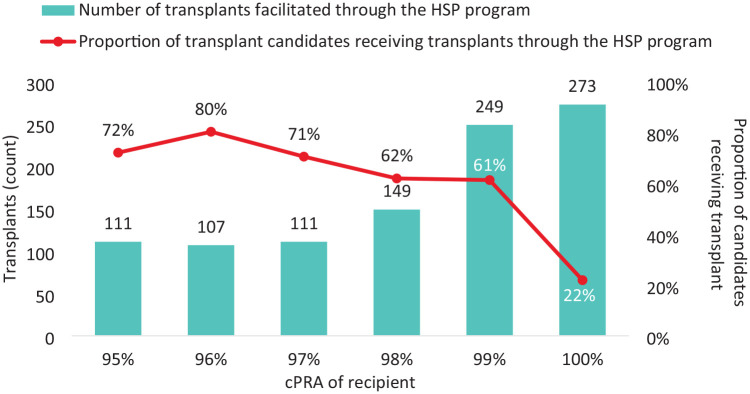
Number of transplants and proportion of transplant candidates receiving transplants through the Canadian HSP Program, by cPRA.

The vast majority of active transplant candidates registered in the Canadian HSP Program at any given time are the hardest to match group (cPRA ≥99%). To date, only 22% of the cPRA 100% transplant candidates have received a transplant (Figure 1).

The median times from first participation in the Program to transplant for recipients with a cPRA of 99% and 100% are significantly longer than for recipients with a cPRA of 95% to 98% recipients (Table 3).

**Table 3.** Time From First Participation in Canadian HSP Program to Transplant for Transplant Recipients.

Recipient cPRA at transplant	Total transplants (n)	Time waiting (days)	
		Range	Median (IQR)
95%	111	2-548	53 (20-116)
96%	107	1-1429	61 (24-181)
97%	111	1-2268	87 (33-180)
98%	149	1-2177	146 (49-311)
99%	249	1-3408	272 (81-584)
100%	273	2-3517	597 (240-1217)

**Figure 2.**
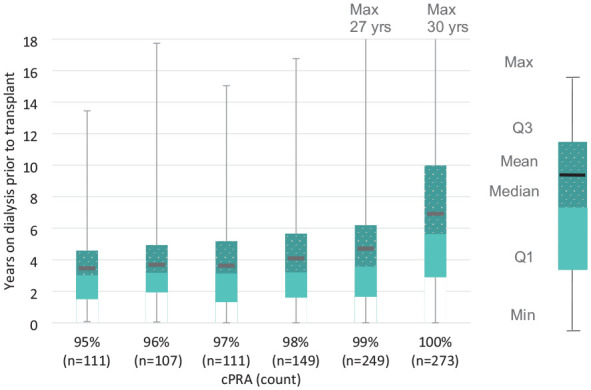
Time on dialysis prior to Canadian HSP Program transplant by cPRA.

**Figure 3.**
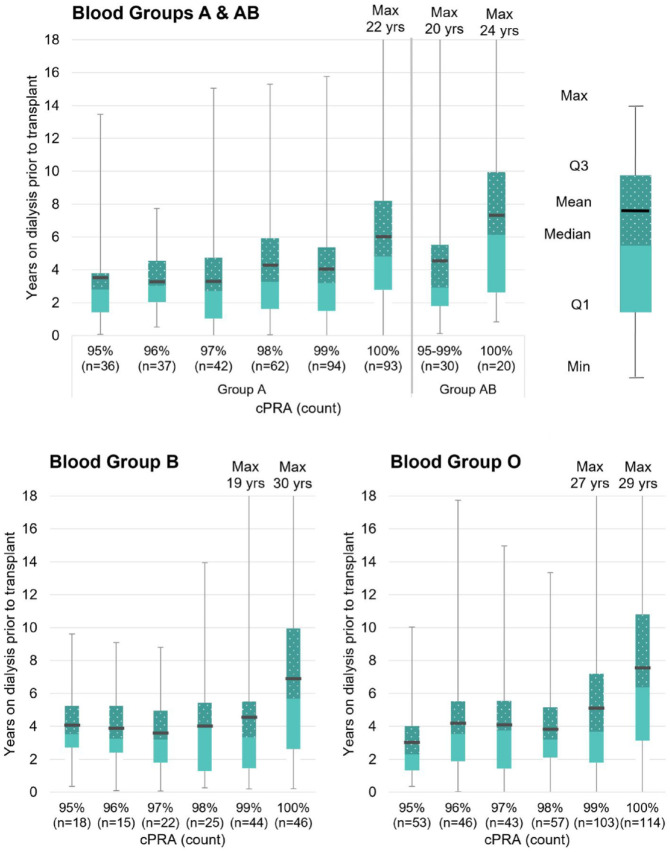
Time on dialysis prior to Canadian HSP Program transplant by blood group and cPRA.

Although patients are required to be on dialysis to be eligible to participate in the Canadian HSP Program, many transplant candidates will begin dialysis well in advance of the first time they are active in the Canadian HSP Program. The median time on dialysis prior to receiving a transplant through the Program is 3.7 years for transplant recipients; however, recipients have been on dialysis for up to 30 years before receiving their Canadian HSP Program transplants (Figure 2). Transplant recipients with a cPRA in the 95% to 98% range waited less than 9 months in the program on average (Table 3) and were on dialysis for between 3 and 4 years on average at the time of transplant (Figure 2). cPRA ≥99% recipients waited 481 days on average longer in the Program than their lower cPRA peers (*t*(968) = 13.6, *P* < .0001). Finally, time on dialysis prior to Canadian HSP Program transplant by blood group and cPRA is presented in Figure 3. HSPs with a cPRA of 100% wait longer regardless of their blood group (Figure 3).

To address these difficulties, the Canadian HSP Program policy on organ allocation was amended in mid-2016 to prioritize very highly sensitized transplant candidates (cPRA ≥99%), which resulted in a significant increase in transplants for these hard-to-match patients. Of the 240 transplants facilitated prior to the implementation of the revised allocation policy, only 51 were to cPRA 100% patients, while 74 transplants were facilitated to cPRA 100% patients in the first 240 transplants that followed the policy’s implementation. This represents a 47% increase in the number of cPRA 100% patients receiving transplants, with the proportion of transplants to cPRA 100% recipients being significantly higher following the implementation of this policy (x^2^(df = 1, N = 474) = 6.291, *P* < .05). Calculated PRA 99% patients who were also given priority access to compatible donors benefited from this policy shift as well.^31^

**Figure 4.**
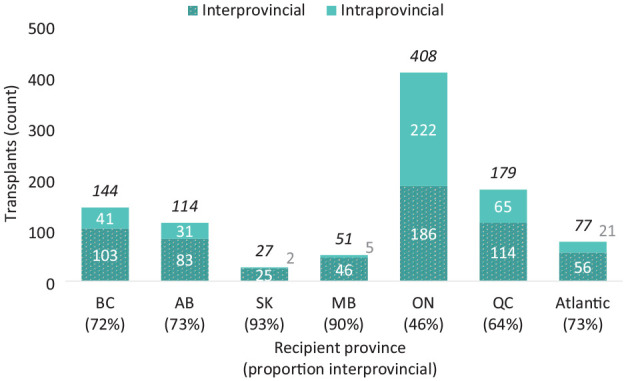
Canadian HSP Program transplants by recipient province and type.

Interprovincial cooperation continues to be crucial to the success of the Canadian HSP Program. Before the end of June 2024, the Canadian HSP Program had facilitated 1000 transplants, 613 of which were interprovincial matches. The proportion of transplants facilitated interprovincially ranges from 46% among Ontario transplant recipients to 93% among Saskatchewan recipients (Figure 4).

More than 40% of Canadian HSP Program transplants have been to Ontario recipients, with just over half of these transplants being intraprovincial. Provinces with smaller populations such as Saskatchewan and Manitoba accounted for a small proportion of Canadian HSP Program activity, with a majority of transplants to recipients in each province other than Ontario being interprovincial.

**Figure 5.**
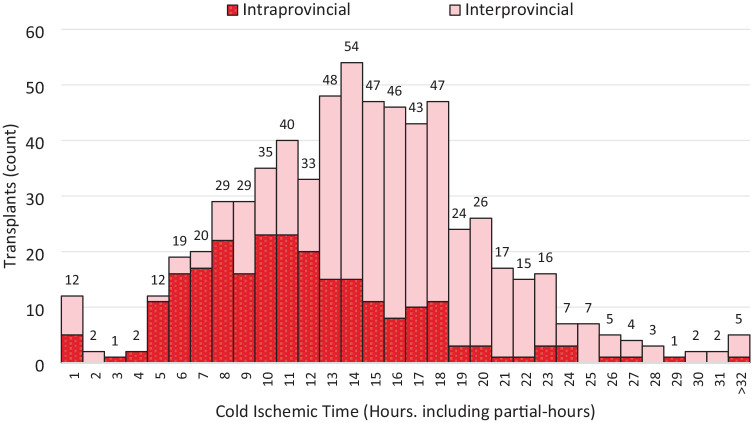
CIT for Canadian HSP Program transplants by intra/interprovincial (n = 653).

Cold ischemic time was available for approximately two thirds (n = 651) of the transplants completed to date (Figure 5). Based on this sample, the median (IQR) CIT is 14 (11-18) hours for all transplants in the sample. As expected, interprovincial transplants have significantly longer median CITs [16 (13-19) hr] than intraprovincial transplants [11 (8-14) hr], with interprovincial median CITs being 5 hours longer (*t*(651) = 10.56, *P* < .0001).

## Discussion

Kidney transplantation (KT) is the optimal treatment for patients with end-stage kidney disease (ESKD), improving the quality of life and survival outcomes.^32^ Highly sensitized patients accrue on the transplant waiting list. Thus, HSPs should be prioritized in kidney allocation systems and linking allocation organizations may increase transplant opportunities for these patients. Access to larger donor pools would also be expected to increase the likelihood of identifying a compatible donor for those who are hard to match.

By the end of June 2024, the Canadian HSP Program achieved the first 1000 transplants. Based on the CTR data, almost half of all transplant candidates were blood group O, with 42% of all O donor kidneys going to blood O recipients. Highly sensitized candidates and recipients were young with an average age of around 50 years old and almost two thirds were female.

Calculated PRA 100% HSPs were the hardest to match. Only one fifth of the registered cPRA 100% candidates received a transplant after a median time of 1.6 years of participating in the Canadian HSP Program. The Canadian HSP Program implemented policy changes to further address the needs of the hardest to match patients, giving priority to cPRA 100% and 99% patients over those with lower cPRAs. Since mid-2016 when the Program allocation criteria changed to prioritize cPRA 100% and 99% HSPs in the Canadian HSP Program, the proportion of transplants directed to cPRA 99% and 100% recipients increased from 44% to 55% (or from 21% to 29% for cPRA 100% recipients specifically) resulted in 47% increased transplant rate.

In the Canadian HSP Program, most donor kidneys were matched interprovincially with more kidneys shipped to smaller provinces resulting in longer CIT (16.2 [SD = 5.5] hours on average for interprovincial transplants, as opposed to 11.5 [SD = 5.3] hours for intraprovincial transplants).

Despite these advancements, current results suggest that fewer transplants may be possible through the Canadian HSP Program in the future, especially for the cPRA ≥99% HSPs (Supplemental Figure S1). This is due to a confluence of factors, including a high proportion of cPRA 100% transplant candidates (the hardest to match) in the candidate pool and an overall decrease in the waitlist size from approximately 500 active patients prior to 2017 to fewer than 400 as of 2021, with active highly sensitized transplant candidates numbering between 350 and 400 from 2021 onward (Supplemental Figure S2). While this can be interpreted as a positive outcome with the Canadian HSP Program contributing to the reduction in HSPs on the waiting list by being able to more efficiently meet the demand for kidney transplantation within this population, 85% of active HSPs are in the cPRA 100% category, the majority of whom will match with fewer than 1 in 1000 donors.

Consequently, and in addition to ongoing monitoring and reporting, proactive measures to enhance transplant opportunities are currently being considered with select measures such as incorporating new antibody crossing options and transitioning to more precise compatibility measurements potentially being implemented in the future. As a complement to ongoing improvements, current operational metrics are extremely positive, including low rates of immunological problems that would prevent proposed transplants from proceeding and infrequent cases in which transplants are not able to be completed to the intended recipient. A major achievement for the Canadian HSP Program is the low number of unexpected positive crossmatches. By the point 1000 transplants had been facilitated through the program, only 20 potential transplants could not be completed because of an unexpected positive crossmatch. An additional 179 potential matches were not able to proceed because of HLA issues identified on review of the offer by the HLA lab Director.

### Post-transplant Outcomes

In order to monitor the quality of transplants facilitated through the Canadian HSP Program, Canadian Blood Services collects and reports on a limited set of key metrics relating to post-transplant outcomes. Data collection is ongoing for the cases that comprise the 1000 Canadian HSP Program transplants, with more time required for data to accumulate to have reliable results that will provide visibility to long-term outcomes. However, preliminary results collected to date suggest that post-transplant outcomes among program recipients are consistent with expected outcomes for patients receiving a deceased donor kidney transplant outside of the Canadian HSP Program.

For instance, based on a sample of 656 Canadian HSP Program transplants that took place between 2014 and 2022 for which data has been collected to date, death-censored graft survival calculated using the Kaplan-Meier product-limit method at 1 year (94.9%), 3 years (90.9%), and 5 years (88.7%) post-transplant among Canadian HSP Program recipients approximated national benchmarks reported by the Canadian Institute of Health Information (93.6%, 87.3%, and 79.8%, respectively, based on mean of annual cohorts reported).^33^ These results, along with results for other metrics such as patient survival and post-transplant rejection episodes, are regularly reviewed by Canadian Blood Services’ Kidney Transplant Advisory Committee as they become available and will hopefully be published in a separate paper in the near future.

More Canadian HSP Program performance metrics can be found on the Canadian HSP Program dashboard (https://profedu.blood.ca/HSP).

### Limitations

Our study limitations include first that it is a retrospective registry data analysis with no available short- and long-term clinical outcomes data at this point (patient and graft survival). Second, given the nature of registry data, not all relevant data may have been captured and reporting may not be complete for all patients. Furthermore, limited data was available for the purposes of this analysis for the period prior to the initiation of the HSP program in Canada and with respect to the success of alternate allocation options in meeting the needs of the highly sensitized population. As such, limited inferences can be made with respect to the specific impact of the program. Nevertheless, it can be reasonably inferred that the vast majority of the transplants facilitated by the program, particularly the interprovincial transplants, would not have been realized in its absence.

In addition, the purpose of this examination being to review the activity of the Canadian HSP program over its more than 10-year history, specific subtopics such as donor availability, offer decision-making, and post-transplant outcomes have not been explored in detail. While topics such as these remain integral to the functioning of the Program, they would be better addressed through separate analyses.

## Conclusion

Since its launch in late 2013, the Canadian HSP Program has successfully facilitated more than 1000 kidney transplants for HSPs who may never otherwise received a transplant through their local provincial waitlists. Sharing access to donors on a national basis has increased transplant opportunities and reduced waitlist times especially for the very highly sensitized (cPRA 99%-100%) Canadian patients. This will likely enhance long-term patient and graft survival with pending results to be analyzed and published in the near future.

## Supplemental Material

sj-jpg-1-cjk-10.1177_20543581241306811 – Supplemental material for Canadian Highly Sensitized Patient Program Report: A 1000 Kidney Transplants Story Supplemental material, sj-jpg-1-cjk-10.1177_20543581241306811 for Canadian Highly Sensitized Patient Program Report: A 1000 Kidney Transplants Story by M. Khaled Shamseddin, Steven Paraskevas, Rahul Mainra, Kyle Maru, Bailey Piggott, Darlene Jagusic, Kathy Yetzer, Lakshman Gunaratnam, Christine Ribic, Joseph Kim, Sunita Singh, Stephanie Hoar, G. V. Ramesh Prasad, Melanie Masse, Isabelle Houde, Myriam Khalili, Kenneth West, Rob Liwski, Sean Martin, Nessa Gogan, Martin Karpinski, Mauricio Monroy-Cuadros, Sita Gourishankar, Olwyn Johnston, James Lan, Christopher Nguen, John Gill and Michel Pâquet in Canadian Journal of Kidney Health and Disease

sj-jpg-2-cjk-10.1177_20543581241306811 – Supplemental material for Canadian Highly Sensitized Patient Program Report: A 1000 Kidney Transplants Story Supplemental material, sj-jpg-2-cjk-10.1177_20543581241306811 for Canadian Highly Sensitized Patient Program Report: A 1000 Kidney Transplants Story by M. Khaled Shamseddin, Steven Paraskevas, Rahul Mainra, Kyle Maru, Bailey Piggott, Darlene Jagusic, Kathy Yetzer, Lakshman Gunaratnam, Christine Ribic, Joseph Kim, Sunita Singh, Stephanie Hoar, G. V. Ramesh Prasad, Melanie Masse, Isabelle Houde, Myriam Khalili, Kenneth West, Rob Liwski, Sean Martin, Nessa Gogan, Martin Karpinski, Mauricio Monroy-Cuadros, Sita Gourishankar, Olwyn Johnston, James Lan, Christopher Nguen, John Gill and Michel Pâquet in Canadian Journal of Kidney Health and Disease
