# Supplementary figures and images for: Canadian Highly Sensitized Patient Program Report: A 1000 Kidney Transplants Story
Source: Can J Kidney Health Dis. 2024 Dec 24;11:20543581241306811. doi: 10.1177/20543581241306811 (PMC11672600; doi:10.1177/20543581241306811)

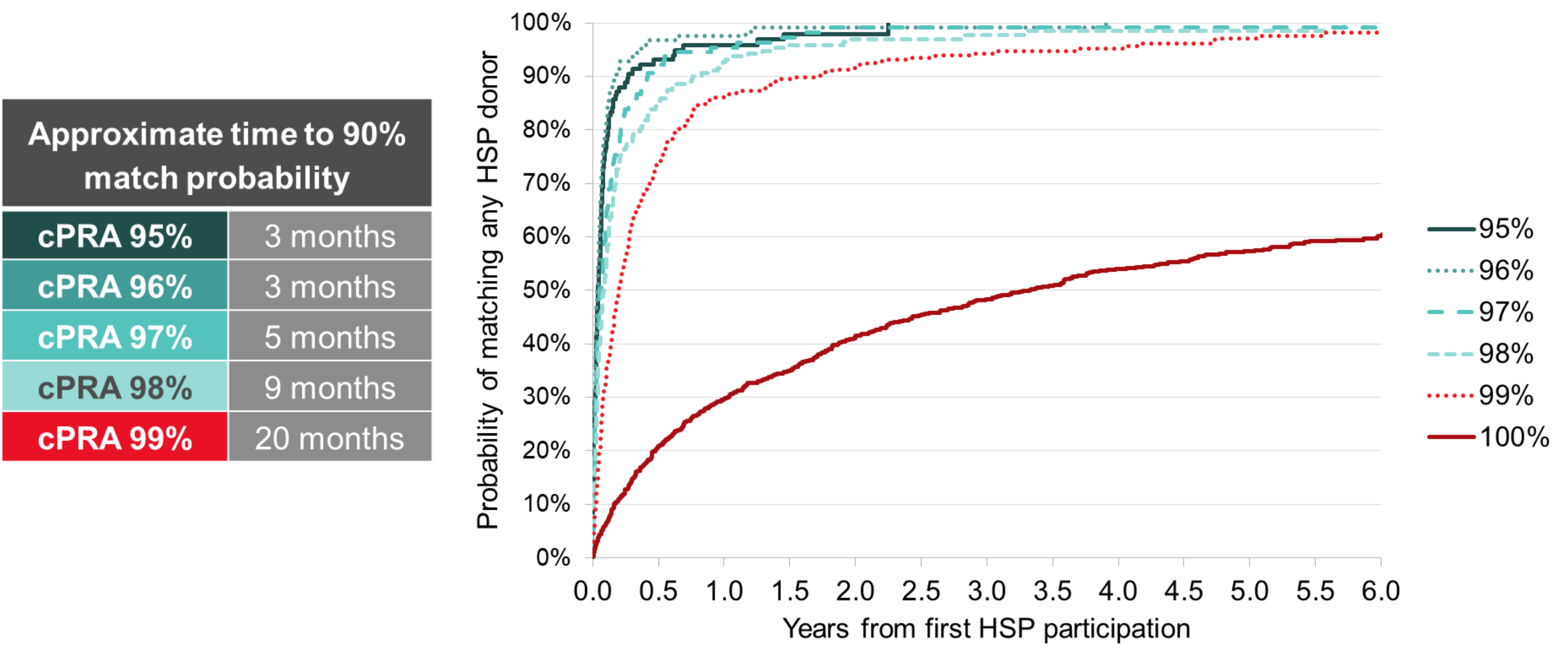

Supplement: sj-jpg-1-cjk-10.1177_20543581241306811 – Supplemental material for Canadian Highly Sensitized Patient Program Report: A 1000 Kidney Transplants Story [file sj-jpg-1-cjk-10.1177_20543581241306811.jpg]

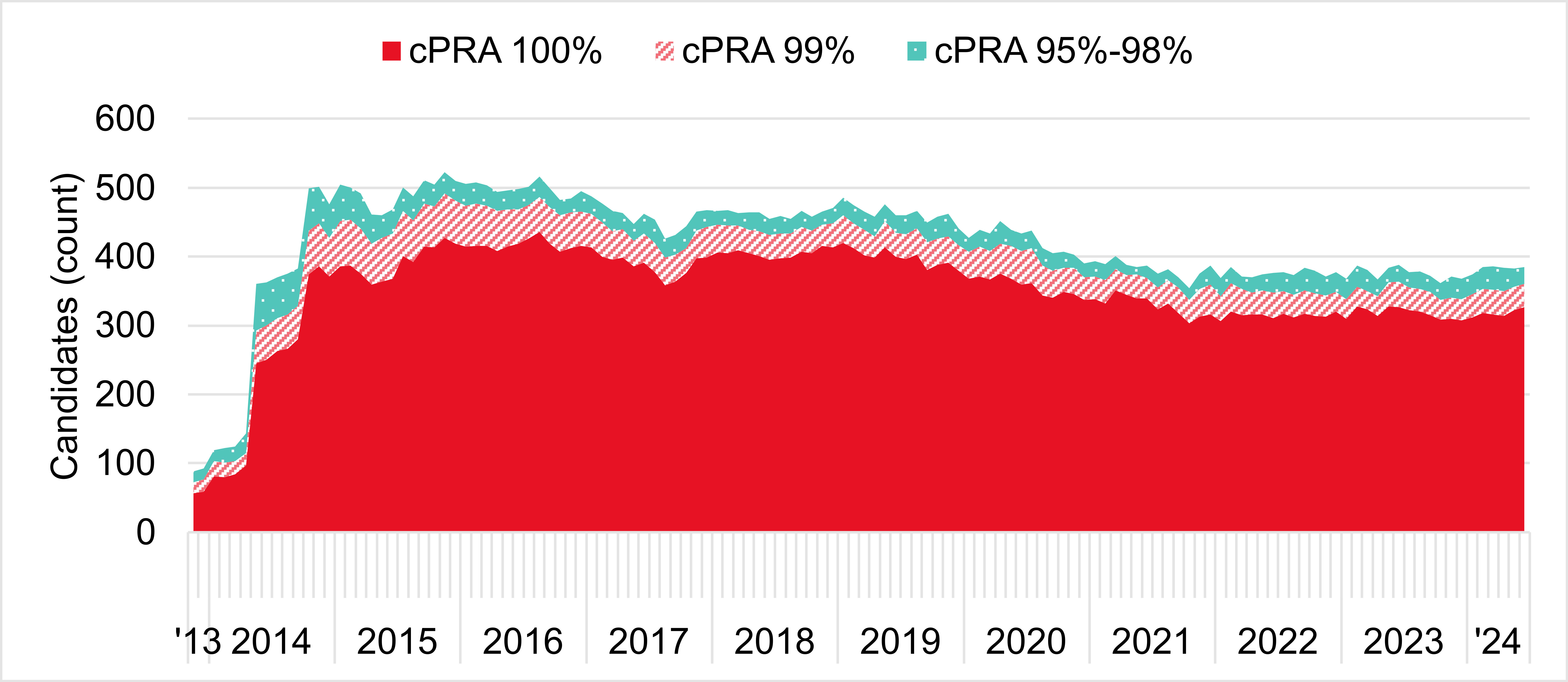

Supplement: sj-jpg-2-cjk-10.1177_20543581241306811 – Supplemental material for Canadian Highly Sensitized Patient Program Report: A 1000 Kidney Transplants Story [file sj-jpg-2-cjk-10.1177_20543581241306811.jpg]
